# Novel object recognition in the dorsomedial and ventral hippocampus of young domestic chicks (*Gallus gallus*)

**DOI:** 10.1007/s00429-026-03078-9

**Published:** 2026-02-02

**Authors:** Anastasia Morandi-Raikova, Alba Cumplido-Mayoral, Uwe Mayer

**Affiliations:** 1 Center for Mind/Brain Sciences (CIMeC), University of Trento, Piazza Manifattura 1, 38068 Rovereto, TN Italy; 2 Department of Cellular, Computational and Integrative Biology (CIBIO), University of Trento, Via Sommarive 9, Povo, 38123 Trento, Italy

**Keywords:** Hippocampal function, Neophobia, Avian cognition, c-Fos, Amygdala (nucleus taeniae), Immediate early genes

## Abstract

The avian hippocampus is well known for its role in spatial cognition, but its contribution to non-spatial domains such as novelty detection remains poorly understood. Here, we examined whether the ventral hippocampus in domestic chicks (*Gallus gallus*) is involved in processing non-spatial novelty. On post-hatching day 5, chicks were exposed to either a novel or a familiar object. Chicks of both sexes recognised the novel object, showing delayed approach, greater distance from the object, and increased locomotion, consistent with neophobic behaviour. Neural activation was mapped using c-Fos immunohistochemistry in the hippocampus, septum, nucleus taeniae of the amygdala (TnA), and intermediate medial mesopallium (IMM). Exposure to novelty selectively increased c-Fos expression in the dorsomedial and ventral hippocampus, independent of hemisphere, while no differences were observed in the septum, TnA, or IMM. This hippocampal activation was significant in males and showed a non-significant trend in females. These findings suggest that the dorsomedial and ventral hippocampus contribute to detecting and evaluating novel stimuli beyond spatial contexts, at least in males, supporting its involvement in novelty-related learning. Together with previous evidence from spatial and social paradigms, these results point to a domain-general role of the avian ventral hippocampus in processing novelty.

**Supplementary Information:**

The online version contains supplementary material available at 10.1007/s00429-026-03078-9.

## Introduction

The hippocampus is a homologous structure in birds and mammals (Striedter 2016), yet its morphology differs markedly across these clades, raising the question of how far their functions are conserved (Bingman and Muzio 2017; Morandi-Raikova and Mayer 2022a; Madison et al. 2024). In birds, the hippocampus occupies the dorsal region of the telencephalon. During mammalian development, the hippocampus also initially forms dorsally but subsequently folds medially between the hemispheres, whereas the avian hippocampus remains in its dorsal position (Medina et al. 2017). Moreover, the avian hippocampus lacks the laminated architecture typical of mammals—although a layered organisation may have been present in their common ancestor (Fujita et al. 2022; Rook et al. 2023). Most research on the avian hippocampus has focused on its role in spatial cognition (reviews: Bingman 2005; Kahn and Bingman 2009; Mayer et al. 2013; Bingman and MacDougall-Shackleton 2017; Sherry et al. 2017; Morandi-Raikova and Mayer 2022a; Ben-Tova and Gutfreund 2022; Gagliardo and Bingman 2024). In contrast, its potential contributions to non-spatial functions remain comparatively underexplored (but see Smulders 2017; Madison et al. 2024), a gap that we address here using the domestic chick (*Gallus gallus*).

Domestic chickens are ground-dwelling birds, and their precocial development makes young chicks a valuable model for studying cognitive functions (Rosa-Salva et al. 2021). They have been used to investigate spatial cognition (Vallortigara and Zanforlin 1986; Vallortigara et al. 1988, 1990; Tommasi and Vallortigara 2000; Pecchia and Vallortigara 2010; Lee et al. 2012; Tommasi et al. 2012; Morandi-Raikova et al. 2020, 2024), complementing research on spatial processing in adult birds (LaDage et al. 2009; Kelly et al. 2010; Cheng et al. 2013; Pritchard et al. 2018; Heinen et al. 2021). Our previous studies examining hippocampal activity in chicks using c-Fos immunohistochemistry revealed notable functional similarities in spatial processing between the chick hippocampus, that of adult birds from other species, and the mammalian hippocampus (Mayer et al. 2016a, 2018; Morandi-Raikova and Mayer 2020, 2021, 2022b; summarised in Morandi-Raikova and Mayer 2022a). Moreover, we found that hippocampal activation increases in response to both novel environments (Morandi-Raikova and Mayer 2020, 2022b) and social encounters with unfamiliar conspecifics (Corrales-Parada et al. 2021). Together, these findings suggest that novelty detection may represent a core, domain-general function of the avian hippocampus extending beyond spatial processing.

Novelty detection plays a crucial role in animal behaviour, enabling organisms to identify and evaluate unfamiliar stimuli in their environment. At the behavioural level, novelty often elicits avoidance responses, such as delayed approach or increased vigilance, a phenomenon commonly referred to as *neophobia*. Neophobia represents the affective and motivational response to novelty and can strongly influence habitat selection, feeding innovation, and adaptation to new resources (Greenberg 1983, 2003; Bertin et al. 2015; Biondi et al. 2010; Sol et al. 2011). Importantly, novelty detection as a cognitive process (i.e., recognising a stimulus as new and distinguishing it from familiar ones) is conceptually distinct from neophobia, although the two are tightly linked in behaviour. Domestic chicks have been used since the early 1990s as a model species to investigate fear- and avoidance-related responses to novelty, particularly in the context of improving welfare in intensively housed poultry (Jones and Waddington 1992). Consistent with this focus, previous avian studies examining the neural basis of neophobic behaviour have primarily targeted fear-related and amygdaloid brain regions (Franchina et al. 1994; Bertin et al. 2015; Perez et al. 2020). In contrast, the potential contribution of the avian hippocampus, widely implicated in novelty processing and contextual evaluation in mammals, has received little attention. In mammals, hippocampal circuits have been shown to regulate behavioural responses to novelty and to gate novelty-induced learning, including through interactions with the lateral septum in the context of social novelties and other limbic structures (Fredes et al. 2021; Rashid et al. 2025). Whether comparable hippocampal mechanisms contribute to the processing and evaluation of non-spatial novelty in birds remains largely unexplored.

Different subdivisions of the avian hippocampus may support distinct functions. Although a consensus on how to define these subdivisions remains elusive (see Morandi-Raikova and Mayer 2022a), recent evidence suggests functional differentiation along all three major axes: anterior–posterior, dorsal–ventral and between the left and right hemispheres. In several bird species, spatially responsive neurons are most abundant in the anterior hippocampus (Agarwal et al. 2023; Payne et al. 2021; Morandi-Raikova and Mayer 2022b), with spatial responses decreasing along the anterior–posterior axis (Payne et al. 2021). This gradient parallels the dorsoventral organisation of spatial coding observed in rodents (Jung et al. 1994), suggesting a possible functional equivalence between the avian anterior–posterior and mammalian dorsoventral axes (Tommasi et al. 2003; Smulders 2017; Morandi-Raikova and Mayer 2021; Payne et al. 2021). Furthermore, functional lateralisation between hemispheres has also been reported in birds, similar to mammals. In chicks, for instance, spatial relational processing is primarily mediated by the right hippocampus (Tommasi et al. 2003; Morandi-Raikova and Mayer 2020, 2021, 2022b). However, the functional distinction between dorsal and ventral hippocampal regions in birds remains poorly understood. Our previous work in chicks showed that the dorsomedial and ventral hippocampus of the right hemisphere responds to unfamiliar conspecifics (Corrales-Parada et al. 2021), while dorsal, dorsomedial, and ventral subdivisions are activated during spatial tasks and exploration of novel environments (Morandi-Raikova and Mayer 2020, 2021, 2022b). Further investigation is therefore required to clarify the specific contributions of the dorsal and dorsomedial/ventral avian hippocampus to spatial and non-spatial behaviours.

The present study investigated the role of the birds’ hippocampus in novelty detection. Specifically, we asked whether the dorsomedial and ventral hippocampus responds to novel objects that are neither social nor spatial in nature. This question builds on our previous findings, which indicate that both social and spatial novelty activate the dorsomedial and ventral hippocampus. We therefore hypothesised that these regions encode novelty per se, independently of stimulus category. To test this hypothesis, chicks were exposed to a novel, non-social object, and their neural activity was compared to that of control chicks familiar with an identical object. Neural activation was assessed using the immediate early gene product c-Fos, a well-established marker of neuronal activity in both mammals and birds (Lanahan and Worley 1998; Tischmeyer and Grimm 1999; Smulders and DeVoogd 2000; Kubik et al. 2007; Golüke et al. 2019; Corrales-Parada et al. 2021).

In addition to the hippocampus, we quantified c-Fos expression in the nucleus taenia of the amygdala (TnA), the septum, and the intermediate medial mesopallium (IMM), which together form key nodes of limbic and associative circuits involved in novelty, affect, and learning in birds. The TnA is the avian homolog of the subpallial medial amygdala in mammals (Cheng et al. 1999; Reiner et al. 2004; Yamamoto et al. 2005; Jarvis et al. 2005) and is implicated in a range of social and affective behaviours, including fear responses (Cohen 1975; Cheng et al. 1999; Absil et al. 2002; Saint-Dizier et al. 2009; Brito et al. 2011; Ikebuchi et al. 2013; Morandi-Raikova and Mayer 2020). Previous studies in chicks reported TnA activation to novel objects (Perez et al. 2020) and environments (Morandi-Raikova and Mayer 2022b), likely reflecting neophobia. We therefore expected the experimental group to show neophobic behaviour and stronger TnA activation than controls. The septum, which receives dense hippocampal input and is a central limbic relay, is known to participate in social behaviours (Goodson et al. 2004a, 2004b; Goodson et al. 2005; Nishizawa et al. 2011; Kelly and Goodson 2014; Wong et al. 2016; Leroy et al. 2018), was also analysed. In our earlier work, the dorsal septum of the left hemisphere responded selectively to unfamiliar conspecifics (Corrales-Parada et al. 2021). We thus hypothesised that septal activity would not differ between groups exposed to non-social objects. Finally, we examined the IMM, a key region for filial imprinting in chicks (Horn 2004; Mayer et al. 2016b). Because all chicks had been imprinted on a familiar object before testing, we expected no differential activation of the IMM between conditions.

## Materials and methods

### Subjects

**Fig. 1:**
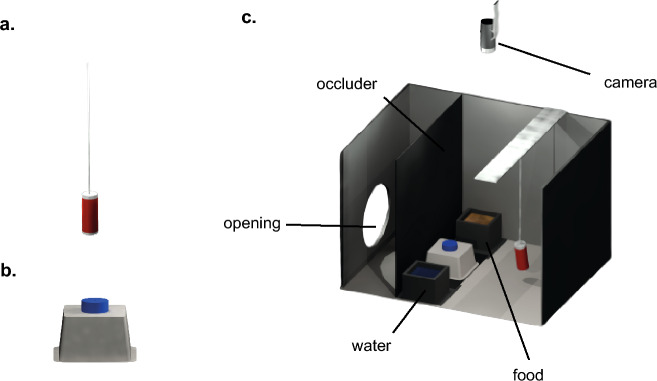
Experimental setup. **a** The imprinting object that chicks were housed with throughout the experiment. **b** The object that was used as the novel stimulus for the experimental group and as the familiar object for the control group. **c** The testing apparatus. Each cage contained food and water ad libitum. The novel or familiar object was introduced through an opening in the black partition wall (occluder), while the experimenter remained unseen

Twenty-eight domestic chicks (*Gallus gallus*; 14 males, 14 females) were used. Fertilised eggs were obtained from a commercial hatchery (CRESCENTI Società Agricola S.r.l.—Allevamento Trepola, code Allevamento127BS105/2) and incubated and hatched in complete darkness at 37.7 °C with 60% humidity. Immediately after hatching, chicks were sexed and housed individually in metal cages (28 × 32 × 40 cm; W × H × L) until post-hatching day 4. Each cage contained an imprinting object—a red cylinder (7.5 × 2 cm; H × D; Fig. 1a), which served solely as a social companion during early development, as is standard practice in chick studies, and was not used as a test stimulus. Food and water were available ad libitum. Cages were maintained at 30–32 °C under a 14:10 h light:dark cycle, with a 30 min transition period between light and dark.

### Experimental setup

Testing was conducted in modified housing cages made of solid metal (28 × 32 × 36 cm; W × H × L, as shown in Fig. 1). Each cage contained a black polypropylene (Poliplak®) partition positioned 10 cm from the front wall (Fig. 1c). This partition had a lower opening (14 × 5 cm; L × H) through which the test object was introduced. The novel object (Fig. 1b) consisted of a blue centrifuge tube cap (Merck, HS4427B; 8 × 8 × 5 cm) glued onto an inverted polystyrene weighing dish (Merck, Z186872; 13.5 × 13.5 × 2 cm) and mounted on an L-shaped black cardboard base (14 × 8 cm). The object was designed to be unfamiliar to the chicks and easily reproducible in other laboratories. Food and water were available ad libitum throughout the testing period. The experimental room was maintained at a temperature of 28–30 °C. Each cage was equipped with an overhead camera (Microsoft LifeCam Cinema for Business) positioned 20 cm above the cage and illuminated by a 25 W warm light lamp placed 37 cm above. The remainder of the room was kept in darkness.

### Testing procedure

On post-hatching day 4, chicks were divided into two groups: “experimental” (n = 14) and “control” (n = 14), each including seven males and seven females. All chicks were placed in the experimental cages with their imprinting objects (Fig. 1c), which remained present during testing but were not involved in the novelty–familiarity manipulation. At this stage, the novel object was absent from the cages of the experimental group; the opening in the *partition wall* was covered with a black polypropylene sheet. In contrast, control cages already contained the test object, which the chicks could freely explore for at least 24 h before testing, making it familiar by the time of the experiment.

Testing took place on post-hatching day 5. For the experimental group, the black cover was removed and the novel object was inserted through the opening, ensuring that the chicks did not see the experimenter’s hand or face. For the control group, the familiar object was briefly removed for a few seconds and reinserted to control for handling effects.

Behaviour was video-recorded starting 15 min before and throughout the 1-h test session. Videos were analysed offline to quantify behavioural responses.

### Behavioural measurements

Videos were analysed blind to the experimental conditions. Video frame size and camera positioning were kept constant across all trials. Cameras were positioned and zoomed in a standardized manner for all recordings, ensuring comparable spatial scaling across cages; therefore, no adjustments to video framing were required. Four dependent variables were manually extracted from these videos: *approach latency*, *number of pecks*, *distance to the object*, and *eye-use index*.

*Approach latency* was defined as the time (in seconds) from the object's introduction into the experimental cage until the chick entered a predefined proximity zone around the object. This zone was defined as one-third of the cage length closest to the stimulus object. To standardise this criterion across videos, a reference line marking this boundary was drawn on a transparent foil fixed to the computer screen. The video was paused when the chick’s head crossed this reference line, and the elapsed time from object introduction to line crossing was recorded as approach latency.

The *number of pecks* represented the total number of pecks directed towards the novel or familiar object during the first 15 min of the test.

The *distance to the object* (in cm) was measured by pausing the video at fixed 60-s intervals during the first 15 min after object introduction (0–15 min). At each time point, the distance from the centre of the chick’s head to the nearest point of the object’s border was measured directly on the screen using a ruler. All measurements were subsequently normalised to the actual dimensions of the experimental cage.

The *eye-use index* measured which eye the chick used to monitor the object. To determine this, a transparent goniometer was placed on the centre of the chick’s head on the computer monitor every 10 s during the first 15 min of the test. At each time point, we recorded the portion of the visual field in which the object was located. The centre of the head and the beak tip were used as reference points to define the 0° line (indicating a perfectly frontal view).

The portions of the visual field corresponding to the binocular frontal region (overall 30°, ranging from 345° to 15°) and the blind spot in the back of the head (overall 60°, ranging from 150° to 210°) were excluded from the analysis (for chick visual fields, see Dharmaretnam and Andrew 1994; Vallortigara et al. 2001). Angles obtained for the left-eye visual field (210°–345°) and right-eye visual field (15°–150°) were averaged across the 15-min observation period. These values were then used to calculate the *Eye-use index* according to the following formula:

$$Eye-use index=\frac{\text{Left eye}}{(\text{Left eye}+\text{Right eye})}$$

Locomotor activity was quantified as the distance travelled by each chick during the 14 min before and 14 min after object introduction, using the automated tracking software *EthoVision* 3.1 (Noldus Information Technology, Leesburg, VA; Noldus et al. 2002). Videos were analysed at a sampling rate of 6 samples per second. The differencing method was used to extract the animals’ position (x, y coordinates). The coordinates obtained in pixels were converted to centimetres by calibrating the software to the width of the experimental cage (28 cm).

### Immunohistochemistry

Brain activity was assessed by quantifying the expression of the immediate early gene (IEG) product c-Fos, using a standardised immunohistochemical protocol previously validated in chickens (Morandi-Raikova and Mayer 2020, 2021, 2022b; Corrales-Parada et al. 2021). All procedures were performed blind to experimental condition.

One hour after the beginning of the test, chicks were euthanised with an intramuscular overdose of 0.4 ml of a 1:1 ketamine (10 mg/ml) and xylazine (2 mg/ml) solution. They were then transcardially perfused with cold phosphate-buffered saline (PBS; 1 mol, pH 7.4, 0.9% NaCl, 4 °C) followed by paraformaldehyde (4% PFA in PBS, 4 °C). Skulls containing the brains were stored in 4% PFA/PBS at 4 °C until processing. Brains were extracted using a stereotaxic head holder (Stoelting), following the procedures described in the chick brain atlas (Kuenzel and Masson 1988) to ensure consistent coronal section orientation (45°). Each brain hemisphere was separated and embedded in 7% gelatine containing egg yolk, then incubated first in 20% sucrose + 4% PFA for 48 h, and subsequently in 30% sucrose + PBS (0.4% PFA).

Each brain hemisphere was cut and stained independently. Four series of 40 μm coronal sections were cut using a cryostat (Leica CM1850 UV) from the posterior one-third of the telencephalon. The first series of sections were used for immunohistochemistry, while the remaining series were stored as backups. Free-floating sections were treated with 0.3% hydrogen peroxide in PBS solution for 20 min to deplete endogenous peroxidase activity. After each step in the protocol, three 5-min washes in PBS were performed. Brain sections were incubated in 3% normal goat serum (S-1000; Vector Laboratories, Burlingame, CA, USA) in PBS for 30 min at room temperature to block nonspecific binding sites. Subsequently, all sections were treated for 48 h at –4 °C with a solution of anti-c-Fos antibody solution (1:1500, rabbit polyclonal, AF-488, Santa Cruz, CA, USA) and then incubated in the secondary antibody solution (biotinylated anti-rabbit made in goat, BA-1000 Vector Laboratories) for 60 min at room temperature. Brain sections were incubated for 75 min at room temperature for signal amplification in the ABC kit (Vectastain Elite ABC Kit, PK 6100; Vector Laboratories). Lastly, VIP kit (SK-4600; Vector Laboratories) was used to visualise the c-Fos-immunoreactive cells, producing a purple-black reaction localised in the nuclei of the activated cells. All stained sections were ordered and mounted with the same orientation for both hemispheres on gelatine-coated slides and counterstained with methyl green (SK-4600; Vector Laboratories), gradually dehydrated in Ethanol and cover-slipped with Eukitt (FLUKA).

### Brain anatomy

**Fig. 2:**
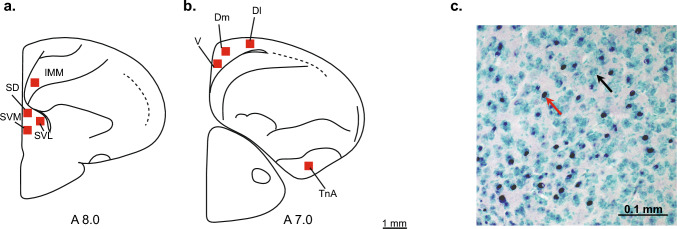
Regions of interest and representative immunohistochemical staining. **a**, **b** Representative placement of cell-counting zones (red squares) within brain areas of interest. Because the chick’s hippocampal formation lacks sharp cytoarchitectonic boundaries, the hippocampal subdivisions shown here represent approximate operational divisions into thirds along the mediolateral axis, with the dorsolateral region overlapping with the parahippocampal areas (APH). The hippocampal formation was partitioned into ventral (V), dorsomedial (DM), and dorsolateral (DL) regions for quantitative analysis. Additional regions of interest included the septum, subdivided into dorsal (SD), ventromedial (SVM), and ventrolateral (SVL) regions; the nucleus taeniae of the amygdala (TnA); and the intermediate medial mesopallium (IMM). **c** Photomicrograph of a hippocampal section from an experimental chick showing c-Fos–immunoreactive cells (black staining; red arrow) and Methyl Green–counterstained cells (light green; black arrow) following immunohistochemical processing

All analyses were performed blind to experimental condition and hemisphere. Following successful immunohistochemical processing, c-Fos-immunoreactive (c-Fos-ir) nuclei appeared purple-black, whereas non-activated cells were light green (Fig. 2c). Photomicrographs of each brain region were taken with a microscope (Zeiss Axio Imager2) equipped with a digital camera (Zeiss AxioCam MRc5) and a 20 × objective with a numerical aperture of 0.5. Exposure time and illumination settings were kept constant across all images. To analyse brain activation in each region of interest, we first identified the area with the highest density of c-Fos-immunoreactive (c-Fos-ir) cells through visual observation under a microscope. A 400 × 400 µm counting frame was then placed over this location, and a cropped image was saved for analysis. The c-Fos-ir cells were counted using the "Analyse Particle" function of ImageJ software (Schneider et al. 2012). A predefined macro converted the images to 8-bit format, set the threshold to 120, adjusted the circularity of particles to range from 0.5 to 1.0, and established the particle size range from 2 to 200.

In this manuscript, we use the term “hippocampus” as a functional shorthand to refer to the hippocampal formation, encompassing hippocampus proper and adjacent parahippocampal regions, unless stated otherwise. For the hippocampus (Hp), eight coronal sections per hemisphere were selected, corresponding to plates A7.8 to A4.6 of the chick brain atlas (Kuenzel and Masson 1988). The hippocampus was operationally subdivided into ventral (V), dorsomedial (DM), and dorsolateral (DL) regions (Fig. 2b). For the septum, five sections per hemisphere were analysed (plates A8.8–A7.6), subdivided into dorsal (SD), ventrolateral (SVL), and ventromedial (SVM) regions (Fig. 2a). For the nucleus taeniae of the amygdala (TnA), five sections per hemisphere were taken from plates A8.8–A6.4 (Fig. 2b). For the intermediate medial mesopallium (IMM), five sections per hemisphere were selected corresponding to plates A9.8–A8.6 (Fig. 2a).

It should be noted that the atlas plates are used here only for anatomical reference, as they do not exactly represent the coordinates in our younger subjects. The Kuenzel and Masson (1988) atlas was based on two-week-old chicks, whereas the present study used five-day-old chicks. For each bird, cell counts were averaged across sections within each area and hemisphere, converted to cell densities (cells/mm^2^), and used for statistical analyses.

### Statistical analysis

We analysed four manually scored behavioural dependent variables—approach latency, number of pecks directed towards the object, distance to the object, and eye-use index—using a two-way MANOVA with Group (control vs. experimental) and Sex (male vs. female) as between-subject factors. Before analysis, assumptions were screened for multivariate normality (Mardia’s test), homogeneity of covariance matrices (Box’s *M*), and homogeneity of variances (Levene’s tests). Because multivariate normality and homogeneity of covariance were violated (Mardia skewness/kurtosis, *p* < 0.001; Box’s *M*χ^2^(30) = 52.82, *p* = 0.006), results are reported using Pillai’s trace. Levene’s tests were nonsignificant for all outcomes (all *p* ≥ 0.26; All assumption checks and full R analysis scripts are provided in the Supplementary Material). Following significant multivariate effects, post hoc univariate ANOVAs were conducted on each dependent variable. Analyses were performed in R using the packages *MVN*, *biotools*, *car*, and *tidyverse*.

We also used automated video tracking (*EthoVision*) to quantify distance moved (cm) during two 14-min phases: Before (pre-novel-object) and After (post-novel-object). For each subject, the total distance per phase was computed by summing the minute bins. Total distances travelled were analysed with a mixed-design ANOVA (Type III sums of squares) with Phase (Before, After) as a within-subject factor and Group (Control, Experimental) and Sex (male, female) as between-subject factors. Planned post hoc tests used estimated marginal means (EMMs) to compare After vs. Before within each Group and Group differences within each Phase, with Bonferroni adjustment.

To characterise the time course of locomotion, a minute-wise repeated-measures ANOVA was fitted with Minute (ranging from − 14 to + 14) as a within-subject factor and Group and Sex as between-subject factors. For multi-level within-subject effects, Greenhouse–Geisser–corrected degrees of freedom and *p*-values are reported. Post hoc comparisons tested for Group differences at each Minute, using Bonferroni-adjusted pairwise contrasts of estimated marginal means. Effect sizes are reported as partial *η*_p_^2^ for ANOVA effects and Cohen’s *d* for post hoc contrasts. Descriptive statistics are given as means ± SEM. All analyses were performed in R (*afex* for ANOVA, *emmeans* for estimated marginal means and post hoc tests, *effectsize* for effect sizes); α = 0.05 (two-tailed).

Differences in c-Fos activation between the experimental and control groups across brain areas were analysed using a repeated-measures ANOVA. The model included two between-subject factors: Group (2 levels: Experimental, Control) and Sex (2 levels: Male, Female); and two within-subject factors: Area (8 levels: HpV, HpDM, HpDL, SD, SVL, SVM, TnA, IMM) and Hemisphere (2 levels: Left, Right). Because residuals of the raw model deviated strongly from normality (Shapiro–Wilk *p* < 0.001), the data were log(x + 10)-transformed, which improved normality (Shapiro–Wilk *p* = 0.022, *W* = 0.99). As Mauchly’s test indicated violations of sphericity for brain areas and its interactions, Greenhouse–Geisser–corrected degrees of freedom are reported. Because the four-way interaction (Sex × Group × Area × Hemisphere) was significant, the data were further analysed separately for males and females using repeated-measures ANOVAs with Group (2 levels: Experimental, Control) as a between-subject factor, and Area (8 levels) and Hemisphere (2 levels) as within-subject factors. Effect sizes are reported as partial *η*^*2*^ (*η*_*p*_^*2*^). Post hoc tests were conducted on the log-transformed data using estimated marginal means (EMMs) with Holm correction for multiple comparisons. In males, group differences were tested within each brain area (averaged across hemispheres). In females, exploratory post hoc tests were used to assess group differences separately for each brain area in each hemisphere. Descriptive results are presented as means ± SEM of the raw (untransformed) data. The raw data were also used to calculate Cohen’s *d* to estimate standardised effect sizes for post hoc contrasts.

Finally, to examine brain–behaviour relationships, we tested whether regional c-Fos density predicted behavioural performance. For each chick, c-Fos-positive cell density (cells/mm^2^) was averaged across hemispheres within each brain area. Pearson correlations were then computed, separately for males and females, between regional c-Fos density and each behavioural measure (approach latency, distance to the object, number of pecks, and eye-use index). For each correlation, we report Pearson’s r, two-tailed p, and sample size (n = 14 per sex). Exploratory correlations in females were also computed separately for left and right hemispheres to test for lateralised relationships between regional activity and behaviour. False discovery rate (FDR) correction (Benjamini–Hochberg) was applied across all region × behaviour tests, and FDR-adjusted p-values (*p*_FDR_) are reported.

All analyses were conducted in R (version 2024.12.0 + 467) using the packages *tidyverse* (data handling and visualisation), *afex* (ANOVA), *emmeans* (estimated marginal means and post hoc tests), and *effectsize* (effect sizes); α = 0.05 (two-tailed). Full R scripts for behavioural and brain analyses are provided as supplementary material.

## Results

### Behavioural results

**Fig. 3:**
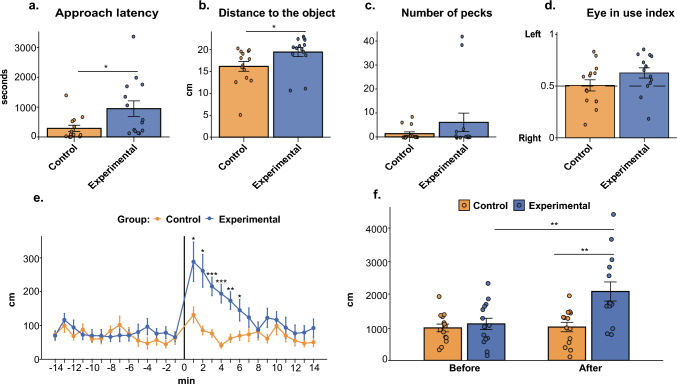
Behavioural responses during the test phase. **a** Approach latency (s) to contact the object; **b** distance to the object (cm) during the test; **c** number of pecks directed toward the object; **d** eye-use index indicating the eye used to inspect the object (1 = left eye, 0 = right eye); **e** distance moved (cm) in fixed 1-min intervals by each group (control: orange; experimental: blue) before and during the test. Because speed is defined as distance divided by time, these per-minute distance measures are a direct estimate of locomotor speed. **f** Total distance moved (cm) before and during the test for each experimental group. Data are presented as mean ± SEM. Dots represent individual values. *p < 0.05, **p < 0.01, ***p < 0.001

Novel object presence affected behaviour (Fig. 3). We first analysed four behavioural dependent variables—approach latency, number of pecks directed towards the object, distance to the object, and eye-use index—across all 28 chicks (n = 7 per Group × Sex). The MANOVA revealed a significant overall effect of Group on the combined behavioural measures (Pillai’s *V* = 0.579, *F*(4,21) = 7.21, p < 0.001). In contrast, there were no significant effects of Sex (*V* = 0.101, *F*(4,21) = 0.59, *p* = 0.674) or Group × Sex interactions (*V* = 0.026, *F*(4,21) = 0.14, *p* = 0.966). Post hoc univariate ANOVAs (Table 1) showed that chicks in the experimental group took longer to approach the novel objects than control chicks, which were familiar with them (mean difference: ~ 661.4 s, *p* = 0.03). The experimental group also maintained greater distances from the objects (mean difference: ~ 3.2 cm, p = 0.044). No significant group differences were found for the number of pecks (*p* = 0.26) or the eye-use index (*p* = 0.116). No Group × Sex interactions were observed for any dependent variable (all *p* ≥ 0.58; Table 1).

**Table 1:** Results of univariate ANOVAs for each behavioural variable

	Control	Experimental	Group	Sex	Group × sex
Approach Latency (s)	287.0 ± 104.9	948.4 ± 260	*F*(1,24) = 5.299; ***p*** = **0.03**; *η*_*p*_^*2*^ = 0.18	*F*(1,24) = 0.760; *p* = 0.392; *η*_*p*_^*2*^ = 0.03	*F*(1,24) = 0.00; *p* = 0.98; *η*_*p*_^*2*^ = 0.00
Distance to Object (cm)	16.2 ± 1.1	19.4 ± 1.1	*F*(1,24) = 4.526; ***p*** = **0.044**; *η*_*p*_^*2*^ = 0.16	*F*(1,24) = 0.727; *p* = 0.402; *η*_*p*_^*2*^ = 0.03	*F*(1,24) = 0.309; *p* = 0.583; *η*_*p*_^*2*^ = 0.01
Eye use Index	0.5 ± 0.1	0.6 ± 0.1	*F*(1,24) = 2.662; *p* = 0.116; *η*_*p*_^*2*^ = 0.10	*F*(1,24) = 1.564; *p* = 0.223; *η*_*p*_^*2*^ = 0.06	*F*(1,24) = 0.161; *p* = 0.692; *η*_*p*_^*2*^ = 0.01
Number of pecks	1.4 ± 0.7	6.1 ± 3.9	F(1,24) = 1.332; *p* = 0.260; *η*_*p*_^*2*^ = 0.05	*F*(1,24) = 0.005; *p* = 0.945; *η*_*p*_^*2*^ = 0.00	*F*(1,24) = 0.001; *p* = 0.972; *η*_*p*_^*2*^ = 0.00

Values are means ± SEM

Columns show main effects and interaction. Significant effects (*p* < 0.05) in bold. Effect sizes are reported as partial *η*^*2*^ (*η*_*p*_^*2*^); values of 0.01, 0.06, and 0.14 correspond to small, medium, and large effects, respectively

The analysis of distance moved across the phases before and after the introduction of the novel or familiar object further confirmed that chicks recognised the novel objects (Fig. 3f). A two-way mixed ANOVA on total distance revealed main effects of Group (*F*(1,24) = 9.24, *p* = 0.006, *η*_*p*_^*2*^ = 0.28) and Phase (*F*(1,24) = 6.51, *p* = 0.017, *η*_*p*_^*2*^ = 0.21). Importantly, a significant Group × Phase interaction was observed (*F*(1, 24) = 5.85, *p* = 0.024, *η*_*p*_^*2*^ = 0.20), indicating that group differences depended on the testing phase. There were no main effects of Sex (*F*(1,24) = 0.23, *p* = 0.637) and no significant interactions involving Sex: Sex × Group (*F*(1,24) = 0.52, *p* = 0.477), Sex × Phase (*F*(1,24) = 0.10, *p* = 0.753), or Sex × Group × Phase (*F*(1,24) = 0.44, *p* = 0.513). Post hoc comparisons showed no group difference during the pre-object phase (Experimental: 1100.3 ± 171 cm; Control: 979.3 ± 116 cm; *t*(24) =  − 0.57, *p* = 0.576). However, after the object was introduced, the Experimental group moved significantly more than the Control group (Experimental: 2077 ± 288 cm; Control: 1005.6 ± 143 cm; *t*(24) =  − 3.25, *p* = 0.004), corresponding to a difference of ~ 1071 cm. Within-group comparisons revealed no change in the Control group across phases (*t*(24) = 0.10, *p* = 0.926), but a significant increase in locomotion (~ 977 cm) in the Experimental group (*t*(24) = 3.52, *p* = 0.002).

Furthermore, a minute-wise repeated-measures ANOVA (Table 2; Greenhouse–Geisser corrected) revealed main effects of Group (*p* = 0.006) and Minute (*p* < 0.001), as well as a significant Group × Minute interaction (*p* < 0.001), indicating that group differences emerged at specific time points. Because distance was quantified in fixed 1-min bins, this measure directly reflects locomotion speed; there were no main effects of Sex and no significant interactions involving Sex (see Table 2). Bonferroni-adjusted post hoc comparisons showed that chicks in the experimental group moved significantly more than controls between minutes 1 and 6 after the object was introduced (all *p* ≤ 0.035; Table 3, Fig. 3e). No group differences were detected at any minute before object introduction, indicating a transient, early post-introduction increase in locomotion in the experimental group.

**Table 2:** Results of the minute-wise repeated measures ANOVA of “distance moved”

Effect	Statistics	Effectsize
Group	*F*(1,24) = 9.236; ***p* = 0.006**	*η*_*p*_^*2*^ = 0.278
Sex	*F*(1,24) = 0.228; *p* = 0.637	*η*_*p*_^*2*^ = 0.009
Group x sex	*F*(1,24) = 0.521; *p* = 0.477	*η*_*p*_^*2*^ = 0.021
Minute	*F*(27,648) = 6.169; *p* < **0.001**	*η*_*p*_^*2*^ = 0.204
Group x minute	*F*(27,648) = 3.833; *p* < **0.001**	*η*_*p*_^*2*^ = 0.138
Sex x xminute	*F*(27,648) = 0.582; *p* = 0.956	*η*_*p*_^*2*^ = 0.024
Group x sex x minute	*F*(27,648) = 0.620; *p* = 0.935	*η*_*p*_^*2*^ = 0.025

Significant effects (*p* < 0.05) in bold

Effect sizes are reported as partial *η*^*2*^ (*η*_*p*_^*2*^); values of 0.01, 0.06, and 0.14 correspond to small, medium, and large effects, respectively

**Table 3:** Results of the minute-wise post hoc analysis of distance moved

Minutes	Control	Experimental	Statistics
-14	72.8 ± 12	70.0 ± 14	*t*(24) = 0.161; *p* = 0.874, *d* = – 0.06
-13	100.2 ± 21	117.0 ± 19	*t*(24) = – 0.587; *p* = 0.563, *d* = 0.23
-12	68.6 ± 11	94.5 ± 22	*t*(24) = – 1.025; *p* = 0.316, *d* = 0.40
-11	88.1 ± 16	72.8 ± 14	*t*(24) = 0.678; *p* = 0.504, *d* = – 0.27
-10	59.7 ± 10	70.1 ± 18	*t* (24) = – 0.505; *p* = 0.618, *d* = 0.20
-9	61.0 ± 15	69.7 ± 18	*t* (24) = – 0.352; *p* = 0.728, *d* = 0.14
-8	84.2 ± 14	66.4 ± 14	*t* (24) = 0.868; *p* = 0.394, *d* = – 0.34
-7	101.9 ± 26	71.0 ± 18	*t* (24) = 0.956; *p* = 0.349, *d* = – 0.37
-6	75.5 ± 19	70.8 ± 17	*t* (24) = 0.182; *p* = 0.857, *d* = – 0.07
-5	55.4 ± 17	80.8 ± 23	*t*(24) = – 0.862; *p* = 0.397, *d* = 0.34
-4	47.1 ± 17	96.7 ± 24	*t*(24) = – 1.662; *p* = 0.11, *d* = 0.64
-3	57.0 ± 15	76.1 ± 15	*t*(24) = – 0.921; *p* = 0.366, *d* = 0.34
-2	44.3 ± 10	78.5 ± 18	*t*(24) = – 1.632; *p* = 0.116, *d* = 0.64
-1	63.5 ± 19	65.8 ± 13	*t*(24) = – 0.106; *p* = 0.916, *d* = 0.04
1	131.7 ± 24	289.4 ± 59	*t*(24) = – 2.437; ***p* = 0.023**, ***d* = 0.95**
2	85.7 ± 13	262.5 ± 48	*t*(24) = – 3.443; ***p* = 0.002**, ***d* = 1.33**
3	76.5 ± 11	216.2 ± 27	*t*(24) = – 4.650; ***p* < 0.001**, ***d* = 1.79**
4	41.1 ± 8	194.8 ± 28	*t*(24) = – 5.164; ***p* < 0.001, *d* = 2.03**
5	62.0 ± 12	173.9 ± 26	*t*(24) = – 3.849; ***p* = 0.001, *d* = 1.46**
6	69.7 ± 14	145.6 ± 32	*t*(24) = – 2.231; ***p* = 0.035, *d* = 0.82**
7	74.0 ± 21	124.1 ± 28	*t*(24) = – 1.459; *p* = 0.158, *d* = 0.54
8	82.0 ± 25	87.5 ± 23	*t*(24) = – 0.156; *p* = 0.878, *d* = 0.06
9	60.8 ± 12	123.0 ± 28	*t*(24) = – 1.987; *p* = 0.058, *d* = 0.76
10	99.5 ± 27	117.2 ± 24	*t*(24) = – 0.491; *p* = 0.628, *d* = 0.19
11	70.0 ± 18	93.8 ± 31	*t*(24) = – 0.648; *p* = 0.523, *d* = 0.25
12	54.9 ± 15	77.0 ± 25	*t*(24) = – 0.741; *p* = 0.466, *d* = 0.29
13	47.3 ± 10	79.5 ± 21	*t*(24) = – 1.361; *p* = 0.186, *d* = 0.53
14	50.4 ± 13	92.7 ± 27	*t*(24) = – 1.392; *p* = 0.177, *d* = 0.54

Bonferroni-adjusted *p*-values are reported, with significant effects (*p* < 0.05) shown in bold

Cohen’s *d* values indicate standardised effect sizes computed from raw data (negative values denote higher movement in the experimental group). Values of 0.2, 0.5, and 0.8 correspond to small, medium, and large effects, respectively

### Brain results

**Fig. 4:**
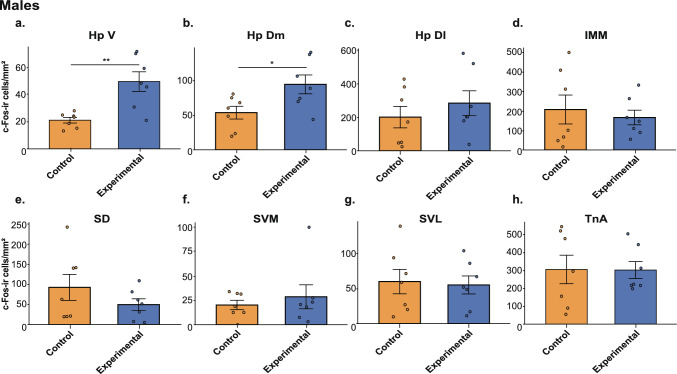
c-Fos–immunoreactive (c-Fos-ir) cell densities across brain areas in male chicks. **a** Ventral hippocampus (HpV), **b** dorsomedial hippocampus (HpDM), **c** dorsolateral hippocampus (HpDL), **d** intermediate medial mesopallium (IMM), **e** dorsal septum (SD), **f** ventromedial septum (SVM), **g** ventrolateral septum (SVL), and **h** nucleus taeniae of the amygdala (TnA). Bars represent mean ± SEM; dots indicate individual values. *p < 0.05, **p < 0.01, ***p < 0.001

**Fig. 5:**
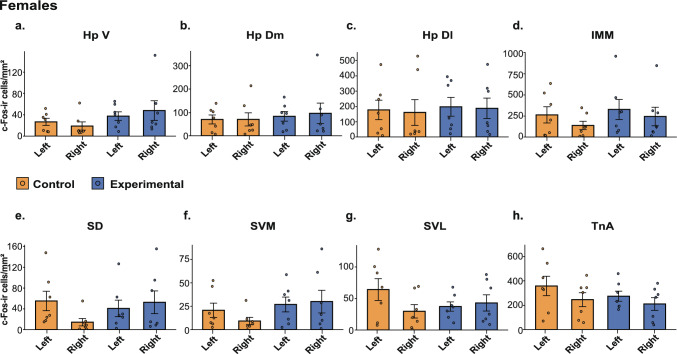
c-Fos–immunoreactive (c-Fos-ir) cell densities across brain areas in the left and right hemispheres of female chicks. **a** Ventral hippocampus (HpV), **b** dorsomedial hippocampus (HpDM), **c** dorsolateral hippocampus (HpDL), **d** intermediate medial mesopallium (IMM), **e** dorsal septum (SD), **f** ventromedial septum (SVM), **g** ventrolateral septum (SVL), and **h** nucleus taeniae of the amygdala (TnA). Bars represent mean ± SEM; dots indicate individual values. *p < 0.05, **p < 0.01, ***p < 0.001

All 28 brains were successfully stained and analysed (Figs. 4 and 5). The repeated-measures ANOVA (Table 4) revealed a significantmain effect of Area (p<0.01) and a significant Hemisphere × Area × Group × Sex interaction (*p* = 0.02), indicating that the pattern of group differences across brain areas differed between males and females and varied by hemisphere. To follow up on the significant four-way interaction in the full model, separate repeated-measures ANOVAs were run for males and females (Table 4). In males, this analysis revealed a significant main effect of Area (*p* < 0.001) and a significant Group × Area interaction (*p* = 0.043), indicating that group differences varied across brain areas. No main effects or interactions involving hemisphere were found. The post-hoc analysis in males revealed a significantly higher c-Fos-ir cell density in the ventral (*p* = 0.001) and dorsomedial (*p* = 0.027) hippocampal formation of the experimental compared to the control group (Fig. 4a, b, Table 5). No significant differences were present in any other brain areas (all *p*-values ≥ 0.354, Table 5).

**Table 4:** Repeated-measures ANOVA summary. Sphericity was assessed with Mauchly’s test

Factors	All data	Males	Females
Group	*F*(1,24) = 0.832; *p* = 0.371; *η*_*p*_^*2*^ = 0.033	*F*(1,12) = 0.376; *p* = 0.551; *η*_*p*_^*2*^ = 0.030	*F*(1,12) = 0.455; *p* = 0.513; *η*_*p*_^*2*^ = 0.037
Sex	*F*(1,24) = 0.550; *p* = 0.466; *η*_*p*_^*2*^ = 0.022	–	–
Sex × group	*F*(1,24) = 0.008; *p* = 0.931; *η*_*p*_^*2*^ = 3.227	–	–
Area	*F*(3.65,87.58) = 101.886; ***p*** < **0.01**; *η*_*p*_^*2*^ = 0.809	*F*(3.48,41.78) = 55.142; ***p*** < **0.001**; *η*_*p*_^*2*^ = 0.821	*F*(2.36,28.27) = 48.556; ***p*** < **0.001**; *η*_*p*_^*2*^ = 0.802
Sex × area	*F*(3.65,87.58) = 1.225; p = 0.306; *η*_*p*_^*2*^ = 0.048	–	–
Group × area	*F*(3.65,87.58) = 2.131; *p* = 0.090; *η*_*p*_^*2*^ = 0.081	*F*(3.48,41.78) = 2.826; ***p*** = **0.043**; *η*_*p*_^*2*^ = 0.191	*F*(2.36,28.27) = 0.876; *p* = 0.443; *η*_*p*_^*2*^ = 0.068
Sex × group × area	*F*(3.65,87.58) = 1.398; *p* = 0.244; *η*_*p*_^*2*^ = 0.055	–	–
Hemisphere	*F*(1,24) = 3.680; *p* = 0.067; *η*_*p*_^*2*^ = 0.132	*F*(1,12) = 0.232; *p* = 0.639; *η*_*p*_^*2*^ = 0.019	*F*(1,12) = 4.079; *p* = 0.066; *η*_*p*_^*2*^ = 0.254
Sex × hemisphere	*F*(1,24) = 1.830; *p* = 0.189; *η*_*p*_^*2*^ = 0.071	–	–
Group × hemisphere	*F*(1,24) = 0.953; *p* = 0.339; *η*_*p*_^*2*^ = 0.038	*F*(1,12) = 0.009; *p* = 0.927; *η*_*p*_^*2*^ = 0.001	*F*(1,12) = 1.295; *p* = 0.277; *η*_*p*_^*2*^ = 0.097
Sex × group × hemisphere	*F*(1,24) = 0.751; *p* = 0.395; *η*_*p*_^*2*^ = 0.03	–	–
Area × hemisphere	*F*(4.80,115.08) = 1.233; *p* = 0.299; *η*_*p*_^*2*^ = 0.048	*F*(3.71,44.47) = 1.548; *p* = 0.208; *η*_*p*_^*2*^ = 0.114	*F*(4.12,49.43) = 1.580; *p* = 0.192; *η*_*p*_^*2*^ = 0.117
Sex × area × hemisphere	*F*(4.80,115.08) = 1.894; *p* = 0.104; *η*_*p*_^*2*^ = 0.073	–	–
Group × area × hemisphere	*F*(4.80,115.08) = 0.674; *p* = 0.638;* η*_*p*_^*2*^ = 0.027	*F*(3.71,44.47) = 1.179; *p* = 0.332; *η*_*p*_^*2*^ = 0.089	*F*(4.12,49.43) = 2.480; *p* = 0.055; *η*_*p*_^*2*^ = 0.171
Sex × group × area × hemisphere	*F*(4.80,115.08) = 2.853; ***p*** = **0.020**; *η*_*p*_^*2*^ = 0.106	–	–

Because several effects involving the brain area violated sphericity and Mauchly’s test can be underpowered, Greenhouse–Geisser–corrected results are reported for all effects that include area. Effect sizes are reported as partial *η*^*2*^ (*η*_*p*_^*2*^); values of 0.01, 0.06, and 0.14 correspond to small, medium, and large effects, respectively

**Table 5:** Post hoc comparisons of c-Fos neural densities between experimental and control groups across brain regions

	Control	Experimental	Statistics
*Males*			
HpV	21.2 ± 2	49.7 ± 7	*t*(12) = 4.115, ***p* = 0.001**, ***d* = – 2.02**
HpDM	53.6 ± 9	94.4 ± 14	*t*(12) = 2.509, ***p* = 0.027**, ***d* = – 1.33**
HpDL	200.1 ± 64	283 ± 74	*t*(12) = 0.964, *p* = 0.354, *d* = – 0.46
SD	92.3 ± 32	49.5 ± 14	*t*(12) = – 0.929, *p* = 0.371, *d* = 0.65
SVM	20.1 ± 5	28.5 ± 12	*t*(12) = 0.269, *p* = 0.792, *d* = – 0.34
SVL	59.9 ± 17	55 ± 13	*t*(12) = – 0.016, *p* = 0.987, *d* = 0.12
IMM	206.9 ± 74	165.9 ± 37	*t*(12) = 0.178, *p* = 0.862, *d* = 0.26
TnA	304.2 ± 79	300.8 ± 47	*t*(12) = 0.559, *p* = 0.587, *d* = 0.02
*Females*			
HpV Left	26.6 ± 7	37.3 ± 8	*t*(12) = 0.964, *p* = 0.354, *d* = – 0.55
HpV Right	18.9 ± 8	47.6 ± 18	*t*(12) = 1.902, *p* = 0.081, *d* = – 0.78
HpDM Left	69.5 ± 19	82.8 ± 21	*t*(12) = 0.522, *p* = 0.611, *d* = – 0.25
HpDM Right	69.9 ± 28	95.8 ± 44	*t*(12) = 0.499, *p* = 0.627, *d* = – 0.27
HpDL Left	176.7 ± 63	196.7 ± 62	*t*(12) = 0.391, *p* = 0.703, *d* = – 0.12
HpDL Right	160.1 ± 84	187.2 ± 67	*t*(12) = 0.704, *p* = 0.495, *d* = – 0.14
SD Left	55 ± 19	40.7 ± 16	*t*(12) = – 0.710, *p* = 0.491, *d* = 0.31
SD Right	14.3 ± 7	52.5 ± 22	*t*(12) = 1.748, *p* = 0.106, *d* = – 0.89
SVM Left	20.7 ± 8	27 ± 8	*t*(12) = 0.568, *p* = 0.581, *d* = – 0.30
SVM Right	9.3 ± 4	30 ± 12	*t*(12) = 1.640, *p* = 0.127, *d* = – 0.87
SVL Left	63.8 ± 17	37 ± 7	*t*(12) = – 0.843, *p* = 0.416, *d* = 0.76
SVL Right	29.5 ± 11	42.7 ± 13	*t*(12) = 0.852, *p* = 0.411, *d* = – 0.43
IMM Left	262.1 ± 96	326.2 ± 119	*t*(12) = 0.712, *p* = 0.490, *d* = – 0.22
IMM Right	137 ± 49	242.7 ± 109	*t*(12) = 0.687, *p* = 0.505, *d* = – 0.47
TnA Left	357.4 ± 80	273.8 ± 42	*t*(12) = – 0.371, *p* = 0.717, *d* = 0.50
TnA Right	245.4 ± 57	209.8 ± 52	*t*(12) = – 0.451, *p* = 0.660, *d* = 0.25

Statistics are Holm-corrected *t*-tests

Values are means ± SEM of raw data (cells/mm^2^)

*Cohen’s d* indicates standardised effect sizes from raw data (negative values denote higher activation in the experimental group); values of 0.2, 0.5, and 0.8 correspond to small, medium, and large effects, respectively

Also, in females, the ANOVA revealed a significant main effect of Area (*p* < 0.001), but no significant interaction between Group and Area (*p* = 0.44). Interestingly, however, a Group × Area × Hemisphere interaction trend was observed (*p* = 0.055), suggesting that in females, group differences may vary by hemisphere in certain regions, although this effect did not reach significance (Table 4; Fig. 5). For exploratory purposes, we also conducted a post hoc analysis in females, which revealed a non-significant trend only in the ventral hippocampus of the right hemisphere (*p* = 0.081). All the other brain areas did not show any differences (all p-values ≥ 0.106).

We next asked whether individual variability in behaviour (latency to approach, distance to the object, pecking, and eye-use bias) was related to neural activity. Pearson correlations between regional c-Fos density and each behavioural measure were computed separately in males and females (n = 14 per sex). No correlation survived FDR correction across brain areas and behavioural variables (all *p*_FDR_ ≥ 0.82; see Supplementary R scripts for full outputs). An exploratory inspection of uncorrected results revealed positive trends between approach latency and c-Fos activity in the septal and hippocampal areas. In females, these involved the right SVM (*r* = 0.60, *p* = 0.023), right SD (*r* = 0.58, *p* = 0.031), and left HpDL (*r* = 0.55, *p* = 0.042). In males, a similar non-significant trend was found between approach latency and HpDL activation (*r* = 0.53, *p* = 0.052). These effects suggest that chicks that hesitated longer to approach tended to show higher activation in regions linked to contextual processing and behavioural inhibition. However, none remained significant after correction and should therefore be considered exploratory.

## Discussion

Our study shows that exposure to a novel object is associated with increased activation of the ventral and dorsomedial hippocampus in male domestic chicks, supporting the hypothesis that these regions contribute to processing non-spatial novelty in birds. Behaviourally, chicks of both sexes recognised the novel object, as indicated by a delayed approach and increased locomotion compared to controls familiar with the same object. Consistent with our hypothesis, exposure to a novel object selectively enhanced c-Fos expression in the dorsomedial and ventral hippocampus of male chicks. This effect was region-specific, independent of hemisphere, and, surprisingly, observed only in males. In females, only non-significant trends were observed in the right ventral hippocampus. No group differences were detected in other hippocampal subdivisions, the septum, or the IMM, and, contrary to our expectations, no effects were found in the TnA.

In a previous study using the same immediate early gene approach, we demonstrated that exposure to unfamiliar conspecifics selectively increased c-Fos expression in the right ventral and dorsomedial hippocampus of domestic chicks, accompanied by enhanced activation in the left dorsal and ventromedial septum (Corrales-Parada et al. 2021). This asymmetric pattern was interpreted as evidence for a lateralised hippocampal–septal circuit engaged in processing socially relevant novelty. In contrast, the present findings show that exposure to a non-social novel object elicits increased activity confined to the ventral and dorsomedial hippocampus in males, with no corresponding involvement of the septum. This pattern suggests that the dorsomedial and ventral hippocampus contribute to novelty detection per se, regardless of whether the stimulus is social or non-social, whereas septal recruitment may be specific to social contexts. Importantly, the increased c-Fos expression observed in both ventral and dorsomedial portions of the hippocampal formation likely reflects the absence of sharp anatomical boundaries between these regions in birds. Rather than discrete subdivisions, the avian hippocampus is characterised by gradual functional transitions, with novelty-related activity decreasing progressively toward the dorsolateral portions. Given that our regional boundaries represent approximate operational divisions, involvement of the dorsomedial hippocampus likely reflects an extension of ventral hippocampal engagement. This interpretation is consistent with our previous findings showing coordinated activation of ventral and dorsomedial hippocampal regions during processing of socially relevant novelty (Corrales-Parada et al. 2021). Taken together, the two studies indicate that the avian hippocampus supports a domain-general mechanism for novelty processing, whose downstream expression depends on the behavioural and affective relevance of the novel.

This interpretation also aligns with our previous studies on spatial novelty in chicks (Mayer et al. 2018; Morandi-Raikova and Mayer 2020, 2022b). In those experiments, we initially analysed the ventral, dorsomedial, and dorsolateral portions of the hippocampal formation separately but subsequently combined them, as no regional differences were detected. The most plausible explanation is that spatial novelty engages both the ventral hippocampus—due to its general sensitivity to novelty—and the dorsal subdivisions, which process spatial information. Supporting this view, studies in zebra finches have shown that during spatial recall, the strongest hippocampal activation differences relative to controls occur in the dorsomedial and dorsolateral regions (Mayer and Bischof 2012). Comparable patterns were observed in an earlier study comparing the learning and recall phases of a spatial memory task in zebra finches (Mayer et al. 2010): higher c-Fos expression during learning reflected broader hippocampal recruitment, whereas recall activity was largely confined to the dorsal portions of the hippocampus (unpublished observations from Mayer and colleagues). Further evidence comes from our recent work comparing chicks that actively explored an environment with those that merely viewed it without exploration (Morandi-Raikova and Mayer 2022b). Active exploration produced stronger activation in the anterior and dorsolateral hippocampus, again suggesting that spatial processing is predominantly localised in these regions. Although the functional predominance of the dorsal hippocampus in spatial tasks warrants further investigation, the overall pattern across studies supports a functional differentiation between the dorsal and ventral hippocampus in birds.

The right-hemispheric dominance observed previously in the ventral and dorsomedial hippocampus during social novelty detection (Corrales-Parada et al. 2021) was not clearly replicated in the present study. Notably, dorsomedial and ventral hippocampal activation in response to non-social novelty was significant only in males and was independent of hemisphere. In females, no significant group effects were detected. Although exploratory analyses revealed a trend toward increased activation in the right ventral hippocampus, this pattern was driven largely by a single individual with a high c-Fos value and should therefore be interpreted with caution. Importantly, the absence of a reliable lateralised effect in females indicates that the present data do not support strong conclusions regarding hemispheric specialisation. Given that males and females were analysed together in our previous study (Corrales-Parada et al. 2021), it remains possible that sex-specific lateralisation effects were obscured. However, resolving this issue will require targeted experiments with larger, sex-balanced samples. At present, our results indicate a robust role of the dorsomedial and ventral hippocampus in novelty processing in males. In contrast, it remains unclear whether the observed sex differences reflect genuine biological variation in hippocampal novelty processing, limited statistical power in females, or methodological factors. Disentangling these possibilities will require dedicated follow-up studies.

Sex differences in neophobia and exploratory behaviour have been frequently reported across avian species. For example, male domestic chicks (*Gallus gallus*) and male blue tits (*Cyanistes caeruleus*) tend to be more neophobic than females when approaching novel food or objects (Jones 1986; Arnold et al. 2007). However, such differences are not universal: in robins (*Erithacus rubecula*) and blackbirds (*Turdus merula*), males and females show comparable responses to novelty (Marples et al. 1998). At the behavioural level, our current study did not reveal any sex differences. Chicks of both sexes displayed behaviour indicative of neophobia, defined as an initial avoidance or delay in approaching unfamiliar stimuli—such as novel objects, food, or environments—reflecting an inhibitory response to novelty rather than mere disinterest (Greenberg 2003; Greggor et al. 2015). Consistent with this interpretation, experimental chicks maintained greater distances from the novel object and took longer to approach it compared to the control group. This pattern aligns with previous reports in domestic chicks (Perez et al. 2020) and house sparrows (*Passer domesticus*) (Kimball et al. 2022), where exposure to novel feeders or food types increased latency to feed. Furthermore, during the first six minutes of testing, experimental chicks exhibited increased locomotion, likely reflecting escape attempts in response to the unfamiliar object before acclimating to its presence—a transient behavioural expression of neophobia.

Importantly, the absence of significant correlations between behavioural measures and hippocampal c-Fos expression after correction for multiple comparisons does not indicate a disconnect between behaviour and neural activation. In the present study, behavioural indices such as approach latency and locomotor activity provide indirect measures of neophobia, reflecting the detection of novelty rather than its neural encoding strength. Motor output and avoidance behaviour are controlled by distributed networks involving multiple brain regions, whereas the hippocampus is thought to contribute primarily to the detection and evaluation of novelty. Accordingly, variability in behavioural expression is not expected to scale linearly with hippocampal activation, and the lack of robust correlations is consistent with a model in which hippocampal novelty processing is upstream of behavioural implementation.

Could the activation of the dorsomedial and ventral hippocampus in males be linked more to neophobia than to novelty itself? These two processes—novelty detection and emotional reactions to novelty—are not mutually exclusive. Novelty detection involves cognitively assessing unfamiliar stimuli and distinguishing between new and known, whereas neophobia reflects the emotional evaluation of that novelty, often manifesting as behavioural inhibition or avoidance (Greggor et al. 2015; Greenberg 2003). The elevated c-Fos levels observed in the dorsomedial and ventral hippocampus of male chicks in our present study, along with the behavioural signs of avoidance and stress-related movement, suggest that this region may contribute to both the identification of novelty and the regulation of the associated affective response. Indeed, evidence from several avian studies supports a role of the hippocampus in anxiety- and stress-related (neophobic) responses (Lemaire et al. 1999; Lormant et al. 2020; Armstrong et al. 2020; for review see Madison et al. 2024).

However, some studies indicate that, particularly, the dorsolateral subdivision of the avian hippocampus is sensitive to stress and may participate in mediating anxiety- or neophobia-related responses. For instance, acute social isolation—an anxiogenic manipulation—elicited strong c-Fos activation in the dorsolateral hippocampus of quail chicks (Takeuchi et al. 1996). In domestic chicks, an effect of an acute stressor on cell proliferation was mainly detected in the dorsolateral portion of the hippocampal formation (Nikolakopoulou et al. 2006). While evidence from pigeons indicates that the ventral hippocampus is more closely tied to learning-related processing than to emotional reactivity per se. Brito et al. (2006) showed that Zenk expression in the ventromedial hippocampus increased specifically when pigeons learned an association between a tone and a shock, but not after exposure to the shock alone. In contrast, dorsal hippocampal activation occurred in all shocked birds, indicating sensitivity to aversive stimulation independent of learning. This dissociation suggests that the ventral hippocampus may integrate novelty with its behavioural relevance—encoding new associations rather than simply signalling emotional arousal. In this context, the dorsomedial and ventral hippocampal activation in males observed in our study likely reflects the chicks’ process of learning about and evaluating the novel object, rather than a purely defensive or fear-based response.

Moreover, recent lesion studies in Japanese quail have provided important insights into functional differentiation within the avian hippocampal formation along the rostrocaudal axis (Damphousse et al. 2022a,b). In particular, lesions encompassing Area V, which overlaps with ventral portions of the hippocampal formation analysed in the present study, were shown to impair spatial but not object memory (Damphousse et al. 2022a). In contrast, lesions targeting more caudal hippocampal regions did not disrupt either spatial or fear-related discrimination (Damphousse et al. 2022b). These findings highlight the complexity of hippocampal contributions to different behavioural domains. Direct comparison with the present results is, however, limited by differences in approach (lesion versus immediate early gene activation), species (quail versus chick), sex (female-only samples versus male-specific effects observed here), and anatomical scope, as our analyses encompassed ventral hippocampal subdivisions across medial and caudal levels rather than a restricted caudal region. Rather than contradicting the present findings, these studies underscore the need to consider sex-, species-, and task-specific factors when interpreting hippocampal function in birds.

Interestingly, we did not observe significant group differences in c-Fos activation within the nucleus taeniae of the amygdala (TnA), despite previous evidence implicating this region in avian fear and neophobic responses (Cheng et al. 1999; Saint-Dizier et al. 2009; Brito et al. 2011; Perez et al. 2020; Morandi-Raikova and Mayer 2020). There are two possible explanations for this discrepancy. One is that the novel object used here may not have been perceived as sufficiently threatening or emotionally salient, but rather as a neutral, unfamiliar stimulus. Alternatively, both groups might have experienced some degree of TnA activation, as our experimental design also included the removal and reintroduction of the familiar object for the control group. Although this manipulation did not elicit behavioural signs of neophobia, it could still have triggered amygdaloid activation. Without a neutral baseline control, we cannot determine whether the control group also showed elevated c-Fos expression.

Lastly, no differences in c-Fos activation were found in the intermediate medial mesopallium (IMM), which aligns with our expectations, as this region was included as a control area. The IMM is a key site for filial imprinting and early learning in chicks (Horn 2004; Mayer et al. 2016b). Because all chicks in our experiment had continuous exposure to their imprinting object from hatching, they were already imprinted by the time of testing. Consequently, no new learning or imprinting-related activation was expected during exposure to either the novel or familiar object. Although prolonged sensory deprivation has been suggested to extend the sensitive period for imprinting, such conditions were not present here, as chicks were continuously exposed to an imprinting stimulus throughout development. The lack of group differences in the IMM thus supports the interpretation that our observed neural effects in other regions, such as the hippocampus, reflect processes explicitly related to novelty detection rather than imprinting mechanisms.

Taken together, our findings identify the ventral hippocampus as a key locus for processing non-spatial novelty in young male chicks. By comparing social, spatial, and object-based contexts across studies, a coherent picture emerges in which the avian hippocampus, and particularly its ventral subdivision, supports a general mechanism for novelty detection. These results contribute to refining our understanding of hippocampal functional organisation in birds and suggest that distinct hippocampal subdivisions may have evolved to flexibly handle the diverse behavioural challenges posed by novel stimuli in different ecological contexts.

## Supplementary Information

Below is the link to the electronic supplementary material.

Supplementary file1 (R 10 KB)

Supplementary file2 (R 20 KB)

Supplementary file3 (CSV 1 KB)

Supplementary file4 (CSV 7 KB)

Supplementary file5 (R 29 KB)

Supplementary file6 (CSV 14 KB)

## Data Availability

The raw datasets used for the statistical analysis are available in the supplement together with the R scripts.
